# Relationship between the Stereocomplex Crystallization Behavior and Mechanical Properties of PLLA/PDLA Blends

**DOI:** 10.3390/polym13111851

**Published:** 2021-06-02

**Authors:** Hye-Seon Park, Chang-Kook Hong

**Affiliations:** Polymer Energy Materials Laboratory, School of Chemical Engineering, Chonnam National University, Gwangju 61186, Korea; parkhseon17@gmail.com

**Keywords:** PLLA, PDLA, homo crystallization, stereocomplex crystallization, mechanical properties

## Abstract

Poly (l-lactic acid) (PLLA) is a promising biomedical polymer material with a wide range of applications. The diverse enantiomeric forms of PLLA provide great opportunities for thermal and mechanical enhancement through stereocomplex formation. The addition of poly (d-lactic acid) (PDLA) as a nucleation agent and the formation of stereocomplex crystallization (SC) have been proven to be an effective method to improve the crystallization and mechanical properties of the PLLA. In this study, PLLA was blended with different amounts of PDLA through a melt blending process and their properties were calculated. The effect of the PDLA on the crystallization behavior, thermal, and mechanical properties of PLLA were investigated systematically by thermogravimetric analysis (TGA), differential scanning calorimetry (DSC), X-ray diffraction (XRD), polarized optical microscopy (POM), dynamic mechanical analysis (DMA), and tensile test. Based on our findings, SC formed easily when PDLA content was increased, and acts as nucleation sites. Both SC and homo crystals (HC) were observed in the PLLA/PDLA blends. As the content of PDLA increased, the degree of crystallization increased, and the mechanical strength also increased.

## 1. Introduction

The poly (l-lactic acid) (PLLA) is a well-known biodegradable and biocompatible polymer that is synthesized from renewable resources [1,2,3,4], as such it has attracted more attention in order to grow its reducibility and eco-friendliness. Additionally, it is biocompatible and non-toxic and therefore it is considered as a favorable material for wide use in biomedical applications such as drug delivery, blood vessel engineering, tissue engineering, and scaffolding. Unfortunately, PLLA application is limited by low melt strength, slow crystallization rate, poor processability, brittleness, low toughness, and low service temperature. Among them, low crystallinity is a major drawback of PLLA application. Blending PLLA with other polymers is a low cost and efficient approach to overcome these limitations and tailoring the properties of the final PLLA-based products for applications [5,6,7,8].

The rate of PLLA degradation is known to be strongly influenced by the degree of its crystallinity [9,10]. Thus, understanding PLLA crystallinity is crucial [11]. Stereocomplex crystallization (SC) between PLLA and its enantiomer poly (d-lactic acid) (PDLA), which was first reported in 1987 by Ikaca et al. [12,13,14,15], provides the most effective and promising method for developing PLLA engineering thermoplastics with superior physicochemical properties. The stereocomplex PLLA/PDLA blend melting temperature (T_m_) was 230 °C, which was 50 °C higher than that of pure PLLA or PDLA, indicating that SC PLA could have better thermal and mechanical properties than pure PLLA [4]. Tsuji and Ikada reported that the tensile strength, stiffness, and heat resistance of stereocomplex PLLA/PDLA blends were much higher than those of pure PLLA or PDLA. Moreover, Brochu et al. proposed that stereocomplex crystallites could act as nucleation sites for homopolymers and accelerate homo crystallization (HC) [16]. Therefore, nucleation efficiency depends on the degree of SC. Research on SC of PLLA/PDLA has attracted much attention. Since it was reported for the first time, the influences of the homopolymer molecular weight [16,17,18], blending ratio [16,17,18,19,20], blending condition [11,12,13,14], and optical purity [16,20] on the formation and properties of the stereocomplex have been widely investigated. The crystal morphology and growth kinetics have also been widely studied [16,17,18,19,20,21,22,23,24]. For instance, Li and co-workers published an excellent review on the recent progress in utilizing SC to enhance the thermal and mechanical properties of PLA [25,26]. Yang and co-workers explained that the mechanism through SC enhanced the strength in PLLA/PDLA blends [27,28,29]. Tan et al. summarized the recent progress in the use of stereocomplexation for the enhancement of PLA thermal and mechanical properties [25]. These fascinating properties enable SC PLLA/PLLA blends to compete with many petroleum-derived engineering plastics in a wide range of fields where material durability and high-performance are paramount such as the aerospace, automotive, and electronic industries.

Polymer blends represent a very important role in the process of new materials, which has better properties in comparison with the net polymers. They are also significant from ecological and economical viewpoints [30]. The polymer mixing properties can be controlled by controlling the morphology for polymer mixing. Compatibility arises from thermodynamic interaction between the blend constituents, which is a function of their physical and chemical structure. Miscible polymer blending, which only involves physical interactions without any complex chemical techniques, constitutes one of the most convenient methods in industrial processing to develop new materials with better physical performances because of the ease of tailoring the glass transition temperature (T_g_), melting temperature, crystallinities, crystalline structure, and morphology [31,32]. The problem of each polymer might be overcome by blending with a different polymer, depending on the end application.

However, most polymer blends are immiscible and because of this drawback, compatibility (reactive or addition) has been demonstrated as the most efficient solution. In this study, reactive compatibilization of the PLLA/PDLA blend was performed, and the thermal, mechanical, and morphological properties of PLLA/PDLA blends with different amounts of PDLA, and compatibilized PLLA/PDLA blends were evaluated.

## 2. Experiments

### 2.1. Materials and Sample Preparation

**Figure 1:**
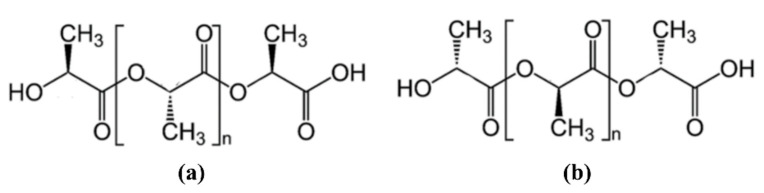
Structure scheme of (**a**) PLLA, (**b**) PDLA.

Polymeric materials such as poly (l-lactic acid) (PLLA, ~260,000 g/mol, CAS No: 81273, Sigma Aldrich, Seoul, Korea) and poly (d-lactic acid) (PDLA, ~20,000 g/mol, PDI ≤ 1.3, CAS No: 767344, Sigma Aldrich, Seoul, Korea) were used in this experiment. The structures of PDLA and PLLA are shown in Figure 1. Prior to melt blending, PLLA and PDLA were dried at 50 °C for 24 h to remove residual moisture. Simple PLLA/PDLA blends with different ratios were prepared using a twin-screw extruder at barrel temperature from 190 °C to 220 °C at 50 rpm rotor speed in an internal mixer (MC5/IM5, Xplore, Suncheon, Korea). For neat PLLA and PDLA, we mixed at 190 °C and 50 rpm. For PLLA/PDLA blends, samples mixed different ratios with 9/1, 8/2, 7/3, 6/4, 5/5 (x/y refers to the blending ratio between PLLA and PDLA) were mixed at 220 °C and 50 rpm. A batch weight of 10 g was maintained as constant in all the different experiments. The total mixing time was fixed at 5 min to prevent PLLA and PDLA thermal degradation in an internal mixer. The films of each sample were prepared by hot press above their melting temperature (190 °C for neat PLLA and PDLA, 225 °C for different blend ratios PLLA/PDLA) under 30 MPa of pressure for 5 min to produce a sheet of size 40 × 40 × 1 mm^3^. The blended samples were cooled to room temperature. All samples were dried at 50 °C for 5 h in an oven before testing.

### 2.2. Thermal Properties

#### 2.2.1. Differential Scanning Calorimeter (DSC)

Thermal properties such as the glass transition temperature (T_g_), melting temperature (T_m_), and percentage crystallinity (X_c_) of the blend samples were determined by differential scanning calorimeter (DSC3, Mettler Toledo, Gwangju, Korea). Scans were carried out in the heating process from −50 °C to 250 °C under a nitrogen atmosphere. The heating rate was set at 5 °C/min. The crystallinity of PLLA in the PLLA/PDLA blends with different phase structures was estimated using DSC. The crystallinity of the PLLA, X_c_ was calculated by the following equation:

(1) $$X_{c}\;\left(\%\right)=\frac{\Delta H_{m}-\Delta H_{c}}{W_{f}\Delta H_{m}^{0}}\times 100$$

where ΔH_m_ and ΔH_c_ are the measured enthalpies of melting and cold crystallization, respectively. $\Delta H_{m}^{0}$ is the enthalpy of pure crystalline PLLA ($\Delta H_{m}^{0}$ = 93.6 J/g) fusion [33] and W_f_ is the weight percent of PLLA in the blends.

#### 2.2.2. Thermogravimetric Analysis (TGA)

Thermogravimetric analysis (TGA2, Mettler Toledo, Gwangju, Korea) was done to assess the thermal stability of the PLLA/PDLA blend samples. The tests were carried out under a nitrogen atmosphere at temperatures of up to 1000 °C at a 10 °C/min heating rate.

### 2.3. Morphological Observations:

#### 2.3.1. X-Ray Diffraction (XRD)

To evaluate the crystalline structure of the blend samples, x-ray diffraction measurements (EMPyrean, PANalytical, Gwangju, Korea) were taken using CuKa radiation in the scattering angle range of 2θ = 0–30° at a scan speed of 4°/min [18].

#### 2.3.2. Polarized Optical Microscope (POM)

The morphologies of the PLLA/PDLA blends with different blend ratios were observed with an optical microscope (Axio Lab.A1, ZEISS, Gwangju, Korea) under crossed polarizers. During preparation, the blended sample was melted on a hot plate (190 °C), pressing it between two coverslips, and quenching to room temperature. Then, the neat PLLA and PDLA were heated at 180 °C for 5 min and the blend samples were heated at 220 °C for 5 min to erase the thermal history of the sample cooled to 140 °C and then quenched.

### 2.4. Mechanical Properties:

#### 2.4.1. Dynamic Mechanical Analysis (DMA)

The dynamic mechanical analysis of the PLLA/PDLA blend samples was performed on a dynamic mechanical analyzer (DMA 2980, TA instruments, Gwangju, Korea). Samples of 13.5~14.0 mm in width and 1.3–1.4 mm in length were tested in single cantilever mode. The sample dimension was entered based on the mean value of five-points. The storage modulus (E’) and tan δ were measured at a 5 °C/min heating rate at a temperature range between −50 °C and 150 °C at a 1 Hz frequency [18].

#### 2.4.2. Tensile Test

The tensile strength, Young’s modulus, and elongation at break were determined with a universal testing machine (5ON, SHIMADZU, Gwangju, Korea) according to ASTM D638. The samples were cut into rectangular strips with dimensions of 1 × 6 cm^2^ and at 23 °C with 50% relative humidity for at least 24 h before testing. Five specimens were tested at a crosshead speed of 1 0 mm/min with a 200 mm initial gap separation [34].

## 3. Result and Discussion

### 3.1. Thermal Properties

**Figure 2:**
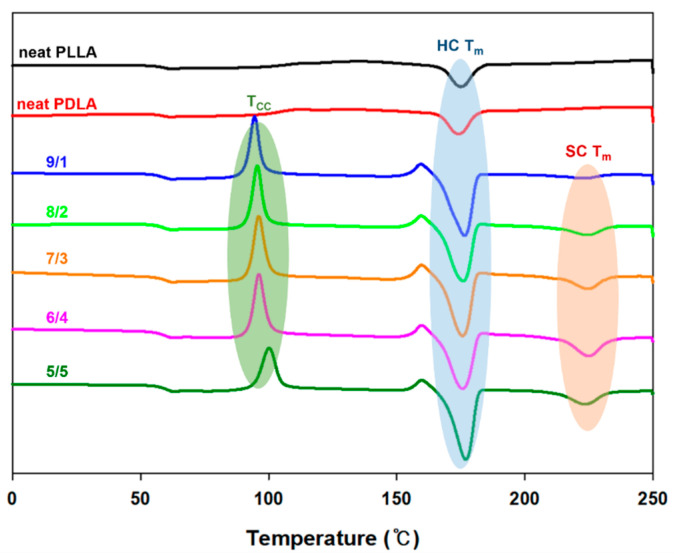
DSC curves of the PLLA/PDLA of different compositions. (x/y refers to the blending ratio between PLLA and PDLA).

The DSC characteristics of PLLA/PDLA blends with different weight ratios are presented in Figure 2 and the corresponding DSC data are listed in Table 1. It can be seen from DSC that the two kinds of polymer are biphasic or homogeneously mixed. The T_g_ values of neat PLLA and PDLA were 58.10℃ and 57.61℃, respectively. PLLA/PDLA blends were one T_g_ peak approximately 58 ℃ for all the samples. The glass transition temperature of the two components is the major factor determining the dynamics and thermodynamic properties [35]. One of the Tg peaks of the blend samples indicates that samples are mixed homogeneously with both polymer matrixes, Figure 2 [36]. Neat PLLA and PDLA polymers had only one T_m_ peak at approximately 175 °C, corresponding to the HC, while there were two T_m_ peaks at 175 °C and 225 °C for the blends with mass ratios of 9/1, 8/2, 7/3, 6/4, and 5/5. A higher T_m_ at approximately 225 °C corresponded to the stereocomplex crystallization (SC) fusion in PLLA/PDLA blends [35,36] due to the simultaneous HC and SC. Moreover, the fact that the T_m_ positions did not shift with different sample compositions indicates that the PLLA/PDLA undergoes stereocomplex and the PLLA undergoes HC almost independently [3]. The high T_m_ at 225 °C indicated the chemical interaction between PLLA and PDLA, and the intermolecular chain mobility between PLLA and PDLA resulted in these high T_m_. Cold crystallinity temperature (T_cc_) is usually located between T_g_ and T_m_. We observed T_cc_ peaks with an increase in PDLA content; these peaks were observed lower in the polymer (chain). The T_cc_ of PLLA/PDLA slightly shifted toward the higher temperature. Since the PDLA has lower mobility than PLLA, we could observe T_cc_ when blending PLLA and PDLA [37].

**Figure 3:**
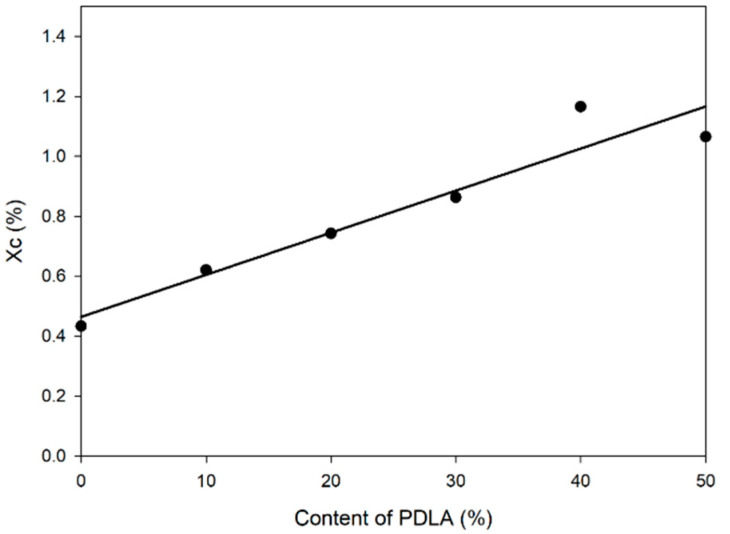
Degree of crystallinity (X_c_) of the PLLA/PDLA blends with different contents of PDLA.

Accurate enthalpy calculations in DSC are important because the degree of crystallinity (X_c_) can be calculated from the enthalpy value of the area under the DSC curve, and the X_c_ for each composition is shown in Figure 3. It is interesting to note that the area of both peaks depends on the PDLA content. The mass ratio 9/1 of the PLLA/PDLA blend showed the smallest crystallization exotherms. The total crystallization enthalpies of SC and HC of the PLLA/PDLA blends with mass ratios of 6/4, 5/5 were 65.44 J/g and 49.86 J/g, respectively. The X_c_ of the blends increased as the content of PDLA increased. Blends with a mass ratio of 6/4 had the highest PLLA crystallization enthalpy. Previous studies have found that the chain mobility of PLLA and PDLA have a significant impact on the competitive crystallization of SC and HC. Because SC crystallization requires alternative folding and packing of PLLA and PDLA chains, it only proceeds when both enantiomeric chains are diffused to the growth fronts [3,31]. Therefore, crystallization increases as the content of PDLA increase. However, in these results, when the blend ratio was PLLA/PDLA (6/4), it showed the highest crystallization than PLLA/PDLA (5/5). The reason for the highest crystallization at 6/4 rather than 5/5 seems to be due to the molecular weight of the PLLA and PDLA polymer. ΔHSC is greatly affected by the optical purity and molecular weight of the blended polymer as molecular weight decreases and optical purity improves, with the crystal phase co-existing SC and HC, and then finally only SC remains, and the overall crystallization is reduced [38,39].

**Figure 4:**
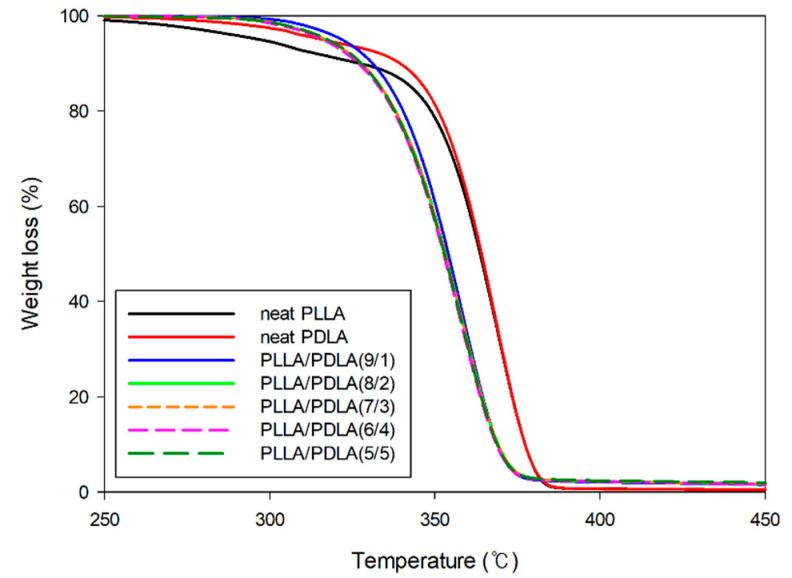
TGA thermograms of the PLLA/PDLA blends with various compositions.

Figure 4 shows the thermal stability of the PLLA, PDLA, and PLLA/PDLA blends. The neat PLLA and PDLA started to decompose at approximately 250 °C, while the PLLA/PDLA blends started to decompose at approximately 300 °C. This indicated that the addition of PDLA in PLLA, which resulted in a PLLA/PLDA blend, lightly increased its onset decomposition temperature and the slight increase in the PLLA/PDLA blends’ thermal stability could have been due to the higher chain entanglement from PLLA and PDLA interaction. Notably, the thermal stability increased as the composition of PDLA increased, but without any significant changes. The properties of PLLA/PDLA blends are largely dependent on the degree of stereocomplexation. In contrast, to the common HC, the enantiomeric PLLA and PDLA chains can be tightly packed side by side in the crystal lattice of SC under the drive of intermolecular hydrogen bonds and dipolar interactions. Because of the denser chain packing as well as the stronger intermolecular interactions, SC imparts substantially enhanced thermal stability and heat resistance [40,41,42].

### 3.2. Morphological Observations

**Figure 5:**
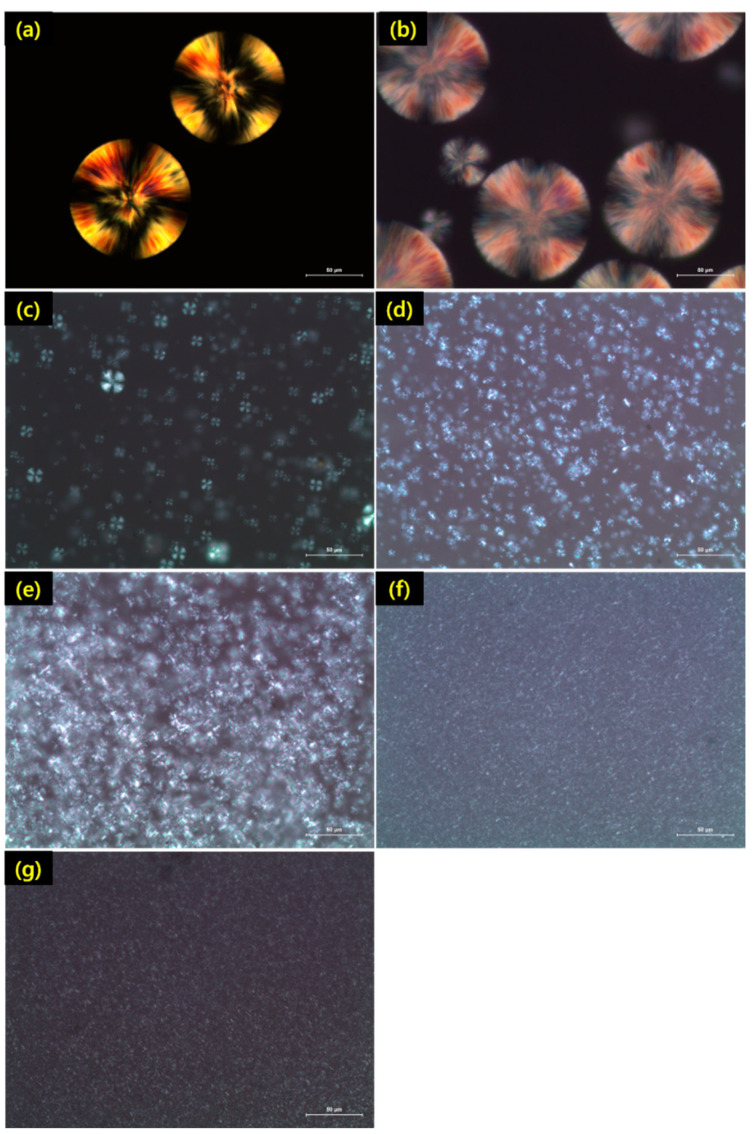
Polarized optical micrograph of PLLA/PDLA blends with different ratio (**a**) neat PLLA, (**b**) neat PDLA, (**c**) PLLA/PDLA (9/1), (**d**) PLLA/PDLA (8/2), (**e**) PLLA/PDLA (7/3), (**f**) PLLA/PDLA (6/4), (**g**) PLLA/PDLA (5/5).

The crystallization process is generally comprised of two stages: nucleation and crystal growth [43]. Figure 5 shows the polarized optical microscopy of the spherulites observed in the blends that contained PDLA after being quenched at 140 °C. The crystallites of the stereocomplex were distributed throughout the PLLA matrix material. The stereocomplex crystallite size ranged from approximately 1 to 25 μm. As shown in Figure 5, there was a gradual increase in the density of the stereocomplex crystallites, consistent with the increased levels of the PDLA in the blends. The size of the spherulites decreased and the number of the spherulites increased significantly with an increase in PDLA content [16,44]. The blends with higher PDLA content had a higher number of nucleation sites. These nucleation sites were SC within conformation and surrounded by the PLLA crystalline phase. However, in blends with higher PDLA content, the spherulites were small in size and the sizes were not uniform. This could be attributed to the simultaneous heterogeneous nucleation by the formed stereocomplex and homogeneous nucleation by the PLLA. When the PDLA content is high, SC is more easily formed and can act as nucleation sites. When the PDLA content is low, PDLA molecules are well distributed in the PLLA matrix and are far apart because of the strong interaction between PLLA and PDLA molecules. Since the PDLA chains have lower mobility than those of PLLA, they can form stereocomplex more easily. These findings are the same as those of the previous section of DSC, where the blends with higher PDLA content heating showed a larger SC.

**Figure 6:**
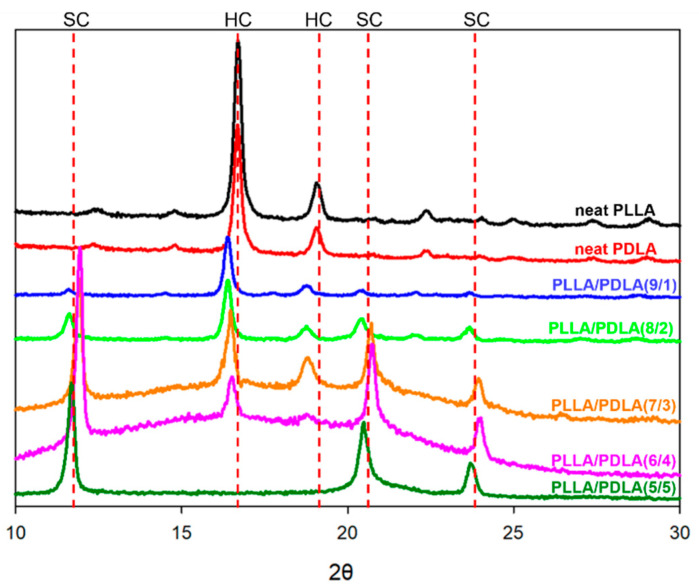
XRD curves of the PLLA/PDLA blends with various compositions.

Similar results were also obtained from the XRD curve. As shown in Figure 6, the diffraction peaks at 16.6° and 18.8° corresponded to (110)/(220) and (203) of PLLA HC, and the diffraction peaks at 11.8°, 20.7°, and 23.9° corresponded to (110), (300)/(030), and (220) of SC [16,39]. The diffraction peak intensity of HC decreased as the ratio of the blend increased, and the diffraction peaks of HC totally disappeared in the blends with a mass ratio of 5/5. The XRD patterns indicated that the samples were completely amorphous prior to rheological characterization in which the XRD results showed PLLA and PDLA segments packed parallel and taking 3_1_ helical conformations [9,45]. The crystallization mechanism can be described by a summary of the above results, where it can be concluded that the PLLA can be crystallized when in exclusively the SC form, while no crystallization behavior was observed for the systems with mixed SC and HC, which is consistent with the results of the PLLA/PDLA blends. Comparing the DSC, POM, and XRD results for PLLA/PDLA blends, we concluded the following. First, the highest PLLA/PDLA blend crystallization could be observed when the mass ratio of PLLA to PDLA was approximately 1(6/4) or 1/1, and when in SC form exclusively. Second, an increase in PDLA content led to an increase in the T_m_ of SC. Third, the highest crystallization enthalpy of SC could be observed during the formation of SC.

### 3.3. Mechanical Properties

**Figure 7:**
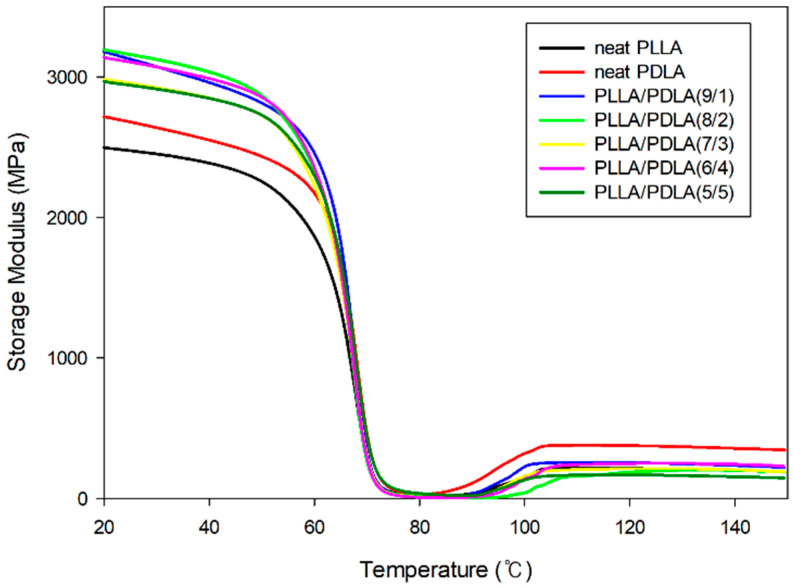
Storage modulus curves with different compositions of PLLA/PDLA dependence of temperature.

Figure 7 shows the temperature dependencies of the storage modulus (E’) for the blends PLLA with PDLA. The storage modulus of the neat PLLA and PDLA gradually decreased from 2531 MPa and 2716 MPa, respectively, at 30 °C to approximately 60 °C and then there was a rapid drop in the glass transition region close to 1 MPa without an increase in temperature. This can be attributed to crystallization. We expected the increased PDLA content to strengthen the PLLA composite and therefore resulted in higher storage values. Apparently, storage modulus is not determined solely by the content of the PDLA, so the propensity of PDLA was also determined to reduce the crystallinity of PLLA. As expected, the E’ plot also highlights the fact that the PLLA/PDLA blends have a higher E’ than neat PLLA and PDLA at 20 °C, indicating an increased stiffness and the E’ increased with an increase in the PDLA content. These results revealed that the intermolecular stereocomplexation had cross-linking points and increased the storage modulus [14]. In particular, PLLA/PDLA blends at ratios of 7/3, 6/4, and 5/5 showed higher storage modulus because of their higher SC.

**Figure 8:**
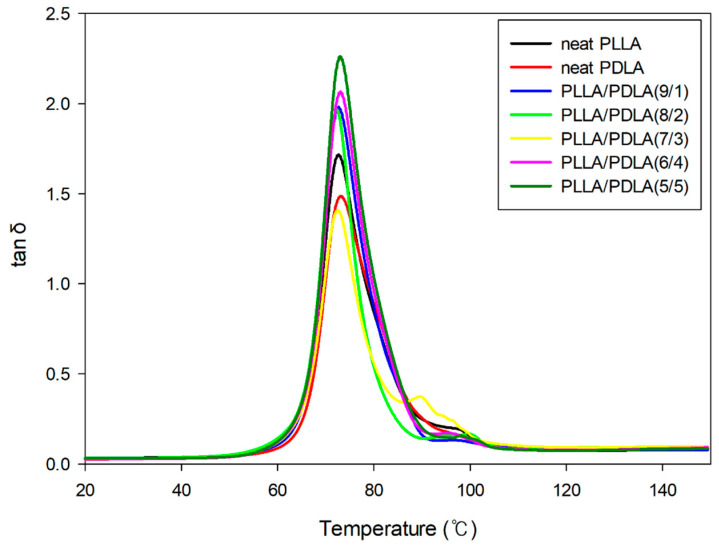
Loss factor tan δ curves with different compositions of PLLA/PDLA dependence of temperature.

As shown in Figure 8, the T_g_ (tan δ peak) peak intensity increased as the crystallinity increased. Therefore, PLLA/PDLA (6/4) had the highest tan δ peak intensity. This trend was similar to that of the DSC results, in which PLLA/PDLA (6/4) had the highest crystallinity. However, tan δ peak intensity was reduced in the PLLA/PDLA (7/3) blend. This could be attributed to the increased free volume in the amorphous domain during the formation of SC in the case there is not enough time for the macromolecular chains to go back to thermodynamic equilibrium.

**Figure 9:**
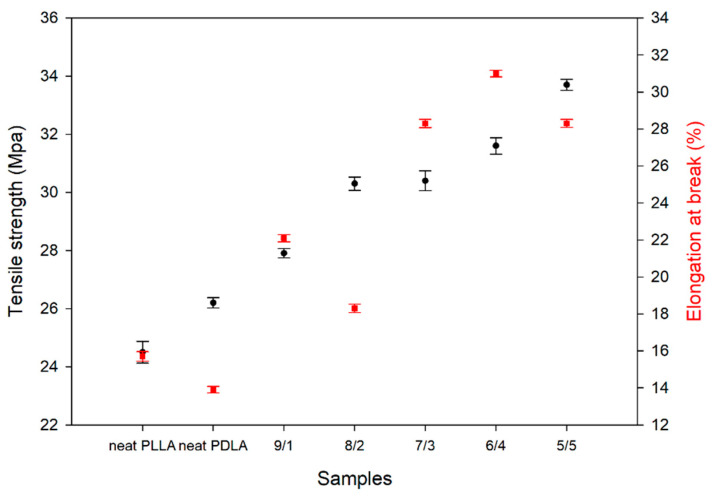
Mechanical properties of the PLLA/PDLA blends with different compositions. (x/y refers to the blending ratio between PLLA and PDLA).

The tensile curve is characteristic of a brittle material, where there is no yield point or plastic deformation. Figure 9 provides the tensile strength and elongation at break curves for corresponding samples of different blending ratios. The yield stress is not given here, as it was comparable with that of tensile strength for all the samples. Neat PDLA had a higher tensile strength (26.2 MPa) than PLLA (24.5 MPa), but there was a small elongation at break (13.9%). The tensile properties of the blend samples were higher than those of neat PLLA and PDLA. The tensile strength of PLLA/PDLA (5/5) was the highest at 33.7 MPa, while elongation at the break was the highest in PLAA/PDLA (6/4). This indicated that the tensile strength gradually increased with an increase in the content of PDLA, but the value of elongation at break did not increase. These results explain the reason why E’ increased; the intermolecular stereocomplexation increased interaction. The formation of the SC structure in the PLLA/PDLA blend samples has a rigid property because numerous material characteristics impact the mechanical properties of polymers such as chemical structure, molecular properties, crystallinity, and molecular orientation. However, the most likely contributor to the slight differences in tensile strength and stiffness is crystallinity. Crystallinity is also known to have a strong influence on elongation.

## 4. Conclusions

The relationship between the crystallization behavior of PLLA and PLDA with SC as well as the thermal and mechanical properties of PLLA and PLLA/PDLA blends were investigated using DSC, POM, XRD, TGA, DMA, and tensile strength. First, neat PLLA and PDLA showed only one T_m_ peak, but PLLA/PDLA blends had two T_m_ peaks. The higher T_m_ peak among the two T_m_ peaks corresponded to SC, and the crystallization behavior of HC and SC occurred independently. Second, exclusively SC could be obtained for the symmetric blends with approximately equal amounts of PLLA and PDLA, but HC and SC coexisted in most of the blends with asymmetric compositions. Third, the formation of SC markedly promoted the crystallinity of PLA. Fourth, the crystallization behavior of PDLA was observed in the following blends: 9/1, 8/2, 7/3, 6/4, and 5/5. The highest amounts of crystallinities were obtained in the blends with approximately equal amounts of PLLA and PDLA, in which there was more SC than HC. Notably, the crystallization behavior of the PLLA blend was determined by the content of PDLA. The stereocomplex crystallite acted as a nucleation site of PLLA and enhanced the crystallization of PLLA significantly. Fifth, as PDLA content increased in the PLLA/PDLA blends, the stiffness increased, which was closely related to SC behavior. From these results, we knew that the mechanical and chemical properties improved as crystallization increased. However, crystallization needs to be controlled for various applications not only in industry, which requires strong mechanical properties, but also in the human body such as blood vessels and tissue engineering, and packaging materials with biodegradability in nature. Polymer materials that can reduce the crystallization of PLLA and control the crystallization will lead to controlling the biodegradation time of the product. We believe that this method could be an open promising approach for its further development.

## Figures and Tables

**Table 1:** Calorimetric parameters characterizing the thermal behavior of different ratio PLLA/PDLA blends.

Samples Name	T_g_ (°C)	T_cc_ (°C)	T_m, HC_ (°C)	T_m, SC_ (°C)	ΔH_HC_ (J/g)	ΔH_SC_ (J/g)
neat PLLA	58.10		175.00		40.56	
neat PDLA	57.61		174.17		32.49	
PLLA/PDLA (9/1)	57.86	94.50	176.50	222.83	48.28	3.99
PLLA/PDLA (8/2)	57.92	95.50	175.83	224.50	45.47	10.16
PLLA/PDLA (7/3)	58.52	96.00	175.83	224.50	44.42	12.14
PLLA/PDLA (6/4)	58.23	96.17	175.82	224.83	44.76	20.68
PLLA/PDLA (5/5)	58.80	100.17	177.17	223.33	37.77	12.09

## Data Availability

The data presented in this study are available on request from the corresponding author.
